# Primary renal lymphoma: a population-based study in the United States, 1980–2013

**DOI:** 10.1038/s41598-019-51635-6

**Published:** 2019-10-22

**Authors:** Jiayuan Chen, Jiangtong Peng, Yongqiang Zheng, Sen Li, Pengcheng Yang, Xiangyi Wu, He Tian, Hui Liu, Shengli Yang, Wenjing Wang, Bian Wu

**Affiliations:** 1 Cancer Center, Union Hospital, Tongji Medical College, Huazhong University of Science and Technology, Wuhan, 430022 China; 2 Department of Cardiology, Union Hospital, Tongji Medical College, Huazhong University of Science and Technology, Wuhan, 430022 China; 3 Department of Urology, Union Hospital, Tongji Medical College, Huazhong University of Science and Technology, Wuhan, 430022 China

**Keywords:** Disease prevention, Cancer, Urological cancer

## Abstract

Primary renal lymphoma (PRL) is a rare lymphoid malignancy with only a few cases reported in the literature. We performed a population-based study of PRL to determine its incidence, clinical characteristics and factors associated with survival using the Surveillance, Epidemiology, and End Results (SEER) database. We identified 723 patients with PRL. The most common histological subtype of PRL was diffuse large B-cell lymphoma (56.3%). The incidence and mortality rate of PRL was 0.053/100,000 person-years and 0.036/100,000 person-years, respectively. The incidence rate of PRL was increasing significantly with an annual percentage change (APC) of 3.45% (*p* < 0.001). The 1-year and 5-year relative survival (RS) rates of patients with PRL were 78% and 64%. The RS of patients diagnosed between 2000 to 2013 was better than that of patients diagnosed between 1980–1999. A multivariate Cox hazards regression analysis revealed that older age, male gender, diagnosis before 2000, advanced stage, not receiving surgical treatment, and DLBCL or T/NK cell lymphoma type were independent predictors of unfavorable survival.

## Introduction

Primary renal lymphoma (PRL) is a rare disease that comprises less than 1% of extranodal lymphomas^1^. It is defined as lymphoma involving the kidney without prior lymphatic disease beyond the kidney^2^. The etiology of PRL is not clear. As a normal kidney does not contain lymphoid tissue^3^, it has been postulated that PRL originates from the renal capsule, which is rich in lymphatic tissue and penetrates the renal parenchyma^4^. Another hypothesis is that lymphoid cells are attracted by the chronic inflammatory conditions of the kidney and develop lymphoma eventually^5,6^. Clinical presentation of PRL is nonspecific and includes such symptoms as gross hematuria, flank pain, loss of weight, fever, or acute/chronic kidney failure^7^. Renal biopsy is the gold standard for diagnosis^8^.

As a result of the rarity of this malignancy, there are very limited data on its incidence, optimal management and clinical outcome, mainly from case reports or case series^9^. Up to our knowledge, only 70 cases were reported as stated in a literature review conducted in 2016^2^. Using data from the Surveillance, Epidemiology, and End Results (SEER) database, our study aimed to examine the incidence, clinical characteristics and survival of patients with PRL as well as to determine prognostic factors in a population-based cohort in the United States.

## Materials and Methods

### Data source

The SEER database is a program of the National Cancer Institute, representing nearly 30% of the US population from 18 registries currently. This population-based program collects information on patient demographics, morphology and site of primary tumor, follow-up and outcomes. Further information about SEER can be found on its web site^10^.

### Study population and variables

**Figure 1:**
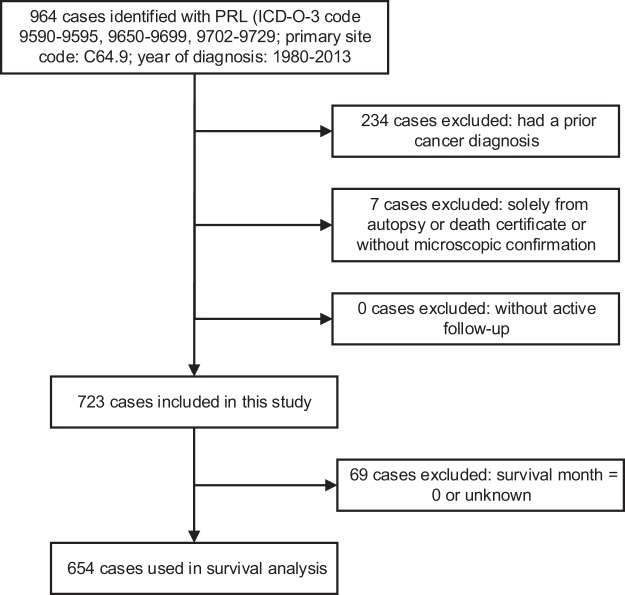
Flow diagram of patient selection within the SEER database between 1980 and 2013.

We obtained clinical data for patients with PRL from the SEER 18 (1973–2013, Nov 2015 Sub) database released in April 2016 using SEER*Stat software (Version 8.3.5; NCI; Bethesda, MD)^11^. We used International Classification of Diseases for Oncology, 3rd edition (ICD-O-3) histologic codes 9590–9595, 9650–9699 and 9702–9729 for lymphoma and primary site code C64.9 for the kidney to identify all patients with PRL diagnosed between 1980 and 2013 (Fig. 1). We excluded patients who had a prior cancer diagnosis, as a prior cancer diagnosis or its treatment might have unknown underlying impacts on outcome of subsequent primary cancer^12^. We also excluded cases without microscopic confirmation or active follow-up (Fig. 1). In addition, we obtained survival data on patients with primary nodal diffuse large B-cell lymphoma (DLBCL) from the same period for comparing survival with primary renal DLBCL^13^. To estimate long-term incidence, we obtained a cohort of patients with PRL from the SEER-9 database from 1980 to 2013^11^.

The following patient-specific information was extracted: age at diagnosis, gender, race, year of diagnosis, marital status, laterality, morphological subtype, cancer stage, therapy type, the length of survival and the cause of death as recorded in the database. Cancer stages reported were according to the Ann Arbor staging system of American Joint Committee on Cancer (AJCC) (7th edition)^14^.

### Statistical analysis

We calculated the long-term incidence rates, mortality rates and corresponding annual percentage changes (APCs) between 1980 and 2013. The incidence rates were age-adjusted to the 2000 US standard population and expressed per 100,000 person-years. A log-linear model was used to calculate APCs of incidence and mortality rates. If there were no cases or deaths recorded in a certain year, the incidence or mortality rate in that year would be zero, which was replaced with a small positive constant (0.0001) for logarithmic transformation^15^. The significance level was calculated using Student’s t test^16^. We analyzed the overall survival (OS), disease-specific survival (DSS) and relative survival (RS) using the Kaplan-Meier method. OS rate is defined as the percentage of survivors (all causes of death) after a duration of follow-up time. DSS rate is defined as the percentage of patients who have not died from PRL (rather than from other causes) in a defined period of time^17^. RS is defined as the ratio of the proportion of observed survivors in a cohort of cancer patients to the proportion of expected survivors in a comparable set of cancer free individuals. The Ederer method was used to calculate RS^18^. The cases whose survival month was 0 or unknown were excluded in the survival analysis (Fig. 1). Univariate and multivariate analysis was done by a Cox proportional hazards model to assess the prognostic effect of the various clinical variables.

All analyses were performed using SEER*Stat software (Version 8.3.5; NCI; Bethesda, MD), Joinpoint Regression Program (version 4.6.0.0; Information Management Services; Calverton, MD), SPSS Statistics software (Version 22; IBM Corporation; Armonk, NY) and STATA MP (version 15.0; StataCorp LP; College Station, TX). Tests were two-tailed, with a p-value of less than 0.05 considered statistically significant.

### Ethical approval

This article does not contain any studies with human participants or animals.

## Results

### Demographic and clinical characteristics

Overall, 723 patients with PRL were identified in the SEER database and were included in the current analysis (Fig. 1). The demographic and clinical characteristics of these patients are shown in Table 1. The mean age at diagnosis was 63.7 (±17.7) years. The majority of the patients were male (62.9%), while the proportion of males in the US general population was 49.1% (Table 1 and Supplementary Table 3). Most patients had a unilateral involvement of the left side (48.3%) or right side (39.1%), while 57 patients (7.9%) had bilateral presentation (Table 1). Patients younger than 18 had a significantly higher proportion (39.1%) of bilateral involvement than adults (6.9%) (*P* < 0.001) (Table S1). A total of 243 patients (33.6%) underwent surgery, and 61 patients (8.4%) received radiotherapy. The preponderance of PRL was non-Hodgkin lymphoma (NHL) (93.2%). The most common NHL subtype identified was DLBCL (56.3%), followed by follicular lymphoma (9.0%) and marginal zone lymphoma (MZL) (7.8%). There were only 5 patients (0.7%) with Hodgkin lymphoma (HL) (Table 1). In total, 423 patients (58.5%) died during the study period. 59.8% of the deaths resulted from PRL. Other major causes of death included cardiovascular diseases (10.9%), other malignant cancers (10.6%), infectious diseases (7.6%) and renal diseases (1.7%) (Supplementary Table 1).

**Table 1:** Demographic and clinical characteristics of patients with primary renal lymphoma (PRL).

Characteristics	No. of Patient (n = 723)
Age	
mean age (±SD)	63.7 (±17.7)
range	2–95
≤60	255 (35.3%)
61–80	369 (51.0%)
>80	99 (13.7%)
Sex	
Female	268 (37.1%)
Male	455 (62.9%)
Race	
White	616 (85.2%)
Black	59 (8.2%)
Asian or Pacific Islander	42 (5.8%)
American Indian	3 (0.4%)
Unknown	3 (0.4%)
Year	
1980–1999	171 (23.7%)
2000–2013	552 (76.4%)
Laterality	
Bilateral	57 (7.9%)
Left	349 (48.3%)
Right	283 (39.1%)
Unknown	34 (4.7%)
Stage	
Stage I	188 (26.0%)
Stage II	178 (24.6%)
Stage III	45 (6.2%)
Stage IV	263 (36.4%)
Unknown	49 (6.8%)
Surgery	
Surgery	243 (33.6%)
Non-surgery	462 (63.9%)
Unknown	18 (2.5%)
Histology	
Hodgkin lymphoma	5 (0.7%)
Non-Hodgkin lymphoma	674 (93.2%)
DLBCL	407 (56.3%)
Follicular lymphoma	65 (9.0%)
Marginal zone lymphoma	56 (7.8%)
Burkitt’s lymphoma	25 (3.5%)
Small lymphatic lymphoma	20 (2.8%)
Continued	
Characteristics	No. of Patient (n = 723)
T/NK cell lymphoma	10 (1.4%)
Non-Hodgkin lymphoma, NOS	91 (12.6%)
Lymphoma, NOS	44 (6.1%)

Note: SD: standard deviation; DLBCL: diffuse large B-cell lymphoma; NOS: not otherwise specified.

### Incidence and mortality

**Figure 2:**
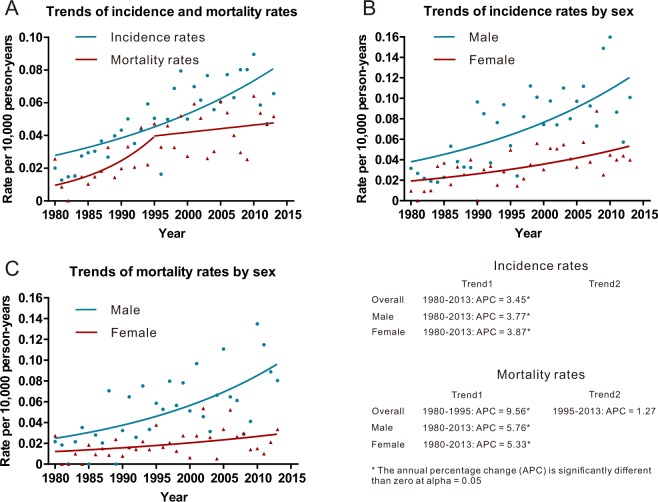
Incidence and mortality rates of primary renal lymphoma (PRL) and annual percentage change (APC) trends of PRL. (**A**) Incidence rates and relative APC trends. (**B**) Mortality rates and APC trends. Notes: incidence and mortality rates were age-adjusted to the 2000 US standard population and expressed per 100,000 person-years.

The age-adjusted incidence and mortality rate during the study period were 0.053 per 100,000 person-years (95% confidence interval [CI], 0.048–0.058) and 0.036 per 100,000 person-years (95% CI, 0.032–0.040), respectively. Overall, the incidence of PRL increased from 0.020 per 100 000 person-years (95% CI, 0.005–0.052) in 1980 to 0.066 per 100 000 person-years (95% CI, 0.041–0.100) in 2013, generating an APC of 3.45% (95% CI, 2.32–4.59; *p* < 0.001) (Fig. 2 and Supplementary Table 4). The mortality due to PRL increased markedly from 1980 to 1995 (APC, 9.59%; 95% CI, 1.35–18.44; *p* = 0.023), and remained stable in the years between 1995 and 2013 (APC, 1.27%; 95% CI, −2.24–4.91; *p* = 0.470) (Fig. 2 and Supplementary Table 5).

The incidence rate of PRL among male population (0.078 per 100,000 person-years; 95% CI, 0.069–0.088) was higher than that of females (0.035 per 100,000 person-years; 95% CI, 0.030–0.041), both of which were increasing significantly in the study period (male: APC = 3.77% [95% CI, 2.26–5.30; *p* < 0.001]; female: APC = 3.87% [95% CI, 0.88–6.94; *p* = 0.009]). Compared to female patients, male patients also had higher mortality rate of PRL (male: 0.059 per 100,000 person-years [95% CI, 0.051–0.068]; female: 0.020 per 100,000 person-years [95% CI, 0.017–0.025]).

### Survival

**Figure 3:**
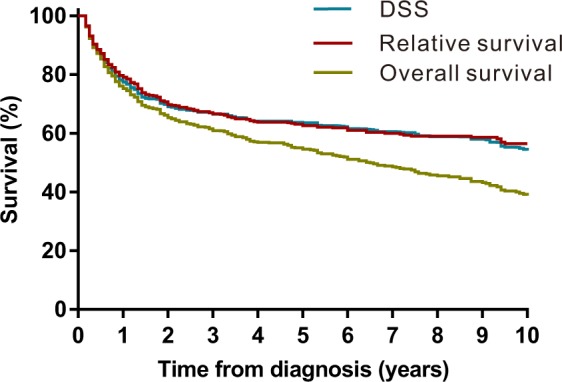
Kaplan–Meier estimate of overall survival (OS), relative survival (RS) and disease-specific survival (DSS) of patients with primary renal lymphoma (PRL).

**Figure 4:**
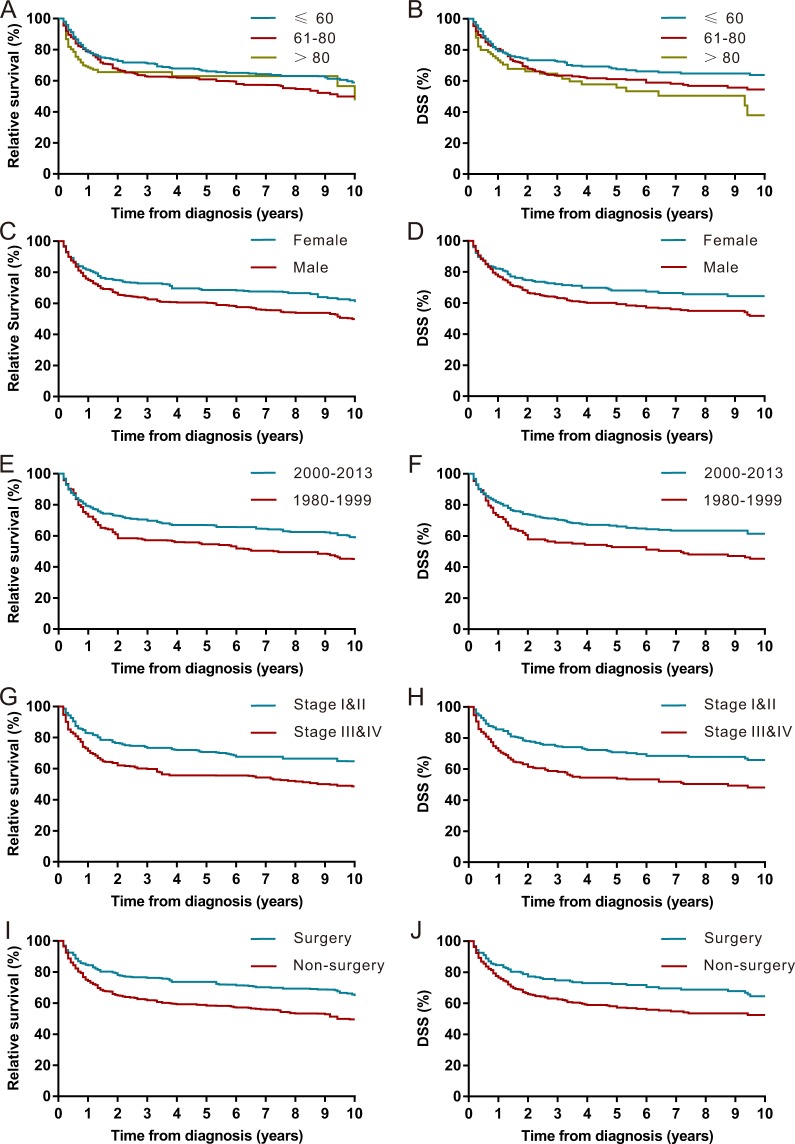
Kaplan–Meier estimate of relative survival (RS) and disease-specific survival (DSS) of patients with primary renal lymphoma (PRL) by different clinical factors. (**A**) RS by age. (**B**) DSS by age. (**C**) RS by gender. (**D**) DSS by gender. (**E**) RS by year of diagnosis. (**F**) DSS by year of diagnosis. (**G**) RS by Ann Arbor Stage. (**H**) DSS by Ann Arbor stage. (**I**) RS by surgery. (**J**) DSS by surgery.

The Kaplan-Meier survival analysis revealed that the RS rates of PRL at 1 year, 5 years and 10 years were 78%, 64% and 55%, respectively (Fig. 3 and Table 2). The RS for patients diagnosed between 2000 to 2013 was better than that of patients diagnosed between 1980–1999 (Fig. 4). A Kaplan-Meier survival analysis also demonstrated a RS and DSS advantage for patients who were younger, were female, had disease of early stage (stage I and II), received surgery and were diagnosed with MZL (Table 2, Fig. 4 and Supplementary Fig. 1).

**Table 2:** Relative Survival (RS) rates and disease-specific survival (DSS) rates of patients with primary renal lymphoma (PRL) for different clinical characteristics.

Variables	RS rates			DSS rates		
	1-year (95% CI)	5-year (95% CI)	10-year (95% CI)	1-year (95% CI)	5-year (95% CI)	10-year (95% CI)
Overall	78% (74–81)	64% (59–68)	55% (34–43)	79% (76–82)	63% (58–67)	57% (52–61)
Age						
≤60	78% (72–83)	66% (59–72)	67% (60–73)	79% (73–84)	68% (61–73)	64% (57–70)
61–80	79% (74–83)	61% (54–67)	61% (54–67)	81% (76–85)	61% (55–67)	55% (48–61)
>80	69% (56–78)	63% (45–77)	63% (45–77)	73% (62–81)	56% (43–66)	38% (21–55)
Sex						
Male	75% (70–79)	60% (54–66)	50% (41–58)	77% (73–81)	59% (54–64)	52% (45–58)
Female	82% (76–86)	69% (61–75)	61% (50–70)	82% (77–87)	68% (61–74)	65% (57–71)
Race						
White	78% (74–82)	65% (59–70)	65% (59–70)	80% (76–83)	63% (59–67)	57% (52–62)
Other	73% (61–81)	59% (45–70)	59% (45–70)	74% (63–82)	59% (47–69)	54% (40–67)
Year of diagnosis						
1980–1999	72% (64–79)	55% (45–63)	45% (35–55)	72% (64–79)	53% (44–61)	45% (37–53)
2000–2013	79% (75–83)	67% (61–72)	58% (49–66)	81% (78–85)	66% (61–70)	61% (56–67)
Laterality						
Right	79% (73–83)	61% (53–68)	49% (38–58)	82% (77–86)	62% (55–68)	54% (46–62)
Left	77% (71–81)	66% (58–72)	59% (48–68)	77% (72–82)	63% (57–68)	57% (50–63)
Bilateral	72% (58–83)	59% (44–72)	56% (38–71)	71% (57–82)	61% (46–73)	58% (42–70)
Stage						
Stage I & II	83% (78–87)	71% (64–77)	65% (55–73)	86% (81–89)	71% (65–76)	66% (59–72)
Stage III & IV	72% (65–77)	56% (49–62)	49% (39–58)	72% (66–77)	54% (47–60)	48% (41–55)
Surgery						
Surgery	84% (78–89)	74% (66–80)	65% (53–74)	85% (79–89)	72% (66–78)	65% (57–71)
Non-surgery	74% (69–78)	58% (52–64)	50% (41–58)	77% (72–80)	57% (52–62)	53% (47–58)
Histology						
Marginal zone lymphoma	94% (79–98)	87% (59–97)	79% (47–93)	98% (87–100)	82% (63–92)	82% (63–92)
Follicular lymphoma	88% (74–95)	71% (53–83)	52% (27–72)	90% (78–95)	70% (56–81)	57% (39–72)
Small lymphatic lymphoma	85% (58–95)	66% (34–85)	48% (12–78)	84% (59–95)	72% (44–87)	60% (28–81)
Burkitt’s lymphoma	78% (54–90)	69% (43–85)	55% (48–63)	77% (54–90)	67% (43–83)	54% (48–60)
DLBCL	71% (65–75)	59% (53–64)	69% (43–85)	73% (68–77)	58% (53–64)	67% (43–83)
T/NK cell lymphoma	51% (23–73)	35% (12–60)	35% (12–60)	57% (28–78)	39% (14–64)	39% (14–64)

Note: RS: relative survival; DSS: disease-specific survival; CI: confidence interval; DLBCL: diffuse large B-cell lymphoma.

**Figure 5:**
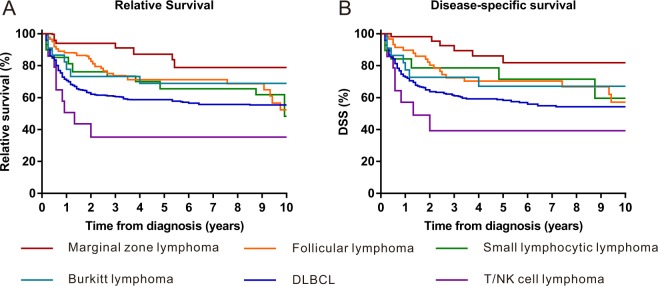
Kaplan–Meier estimate of relative survival (RS) and disease-specific survival (DSS) of patients with primary renal lymphoma (PRL) by histological subtype. (**A**) RS. (**B**) DSS.

As the most prevalent subtype, the estimated 1-year, 5-year and 10-year RS rates for DLBCL were 71%, 59% and 55%, respectively (Table 2, Fig. 5). Compared with nodal DLBCL, we did not find a survival advantage for renal DLBCL (Supplementary Fig. 2).

In the univariable analysis, older age (>80 years old vs <60 years old: HR = 2.98, *p* < 0.001), male gender (HR = 1.33, *p* = 0.012), diagnosis before 2000 (HR = 1.33, *p* = 0.014), advanced stage (HR = 1.40, *p* < 0.001), not receiving treatment with surgery (HR = 1.41, *p* = 0.003) and a histologic subtype of DLBCL (HR = 2.24, *p* = 0.005) were associated with unfavorable survival for patients with PRL (Table 3). The results of multivariable analysis further demonstrated the correlations between worse survival for patients with PRL and older age (>80 years old vs <60 years old: HR = 3.34, *p* < 0.001), male gender (HR = 1.38, *p* = 0.005), diagnosis before 2000 (HR = 1.40, *p* = 0.007), advanced stage (HR = 1.39, *p* = 0.004), not receiving treatment with surgery (HR = 1.50, *p* = 0.001) and the histologic subtype of DLBCL (HR = 2.48, *p* = 0.002) or T/NK cell lymphoma (HR = 4.37, *p* = 0.021) (Table 3).

**Table 3:** Univariate and multivariate Cox analysis of survival in patients with primary renal lymphoma (PRL) by different clinical variables.

Variables	Univariate		Multivariate	
	HR	*p*	HR	*p*
Age				
≤60				
61–80	1.86 (1.46–2.38)	<0.001	1.90 (1.47–2.44)	<0.001
>80	2.98 (2.13–4.18)	<0.001	3.34 (2.34–4.77)	<0.001
Sex				
Female				
Male	1.33 (1.06–1.65)	0.012	1.38 (1.10–1.73)	0.005
Race				
White				
Other	0.92 (0.66–1.27)	0.598	1.19 (0.85–1.67)	0.314
Unknown	1.05 (0.14–7.51)	0.958	0.84 (0.12–6.13)	0.864
Year				
1980–1999	1.33 (1.06–1.68)	0.014	1.40 (1.10–1.80)	0.007
2000–2013				
Laterality				
Right				
Left	0.89 (0.71–1.10)	0.282	0.89 (0.71–1.11)	0.311
Bilateral	0.79 (0.53–1.18)	0.250	0.78 (0.51–1.19)	0.247
Unknown	0.66 (0.35–1.26)	0.206	0.58 (0.29–1.13)	0.111
Stage				
Stage I & II				
Stage III & IV	1.40 (1.12–1.74)	<0.001	1.39 (1.11–1.75)	0.004
Unknown	1.95 (1.35–2.81)	<0.001	1.67 (1.14–2.47)	0.009
Surgery				
Surgery				
Non-surgery	1.41 (1.13–1.76)	0.003	1.50 (1.18–1.91)	0.001
Unknown	1.49 (0.83–2.65)	0.181	1.46 (0.79–2.70)	0.224
Histology				
Marginal zone lymphoma				
Follicular lymphoma	2.01 (1.06–3.79)	0.032	1.97 (1.04–3.73)	0.039
Small lymphatic lymphoma	2.03 (0.93–4.47)	0.076	2.21 (1.00–4.89)	0.051
Burkitt’s lymphoma	1.18 (0.49–2.86)	0.708	1.51 (0.60–3.77)	0.381
DLBCL	2.24 (1.28–3.94)	0.005	2.48 (1.40–4.38)	0.002
T/NK cell lymphoma	3.03 (0.99–9.29)	0.053	4.37 (1.40–13.57)	0.011
Other	2.18 (1.21–3.93)	0.010	2.04 (1.11–3.73)	0.021

Note: HR: Hazard ratios; CI: confidence interval; DLBCL: diffuse large B-cell lymphoma; *Included lymphoma, NOS (not otherwise specified); non-Hodgkin lymphoma, NOS and Hodgkin lymphoma.

## Discussion

In this study, we performed an analysis of a population-based cohort of patients with a diagnosis of PRL as a first primary cancer in the US National Cancer Institute database. To our knowledge, this is the largest evaluation of PRL to date.

The very existence of PRL has been controversial. In recent years, increasing case reports of PRL have confirmed the presence of the disease^2,19–23^. We also observed an increased incidence rate of PRL from 1980 to 2013 in both male and female patients. Further investigation is required to discern whether our reported increase in PRL incidence is true or related to an increase in the recognition of this disease as an entity over time^2,24^.

The median OS of patients with PRL that we calculated was 77 months, which is better than the OS found by a previous review of 49 patients^2^. We observed a discrepancy in survival by gender in our analysis. Females had better prognoses than males. In the Cox proportional hazards model, age greater than 60 years was associated with a poor prognosis. This association is well established in NHL as 60 years is the cutoff value as a component of the International Prognostic Index (IPI)^25^. In our model, Ann Arbor stage was also a determinant.

An improvement in prognosis after the year 2000 was also noted. We observed increased 5-year RS and DSS rates since 2000. We also found that both the incidence and mortality rates elevated with time. However, the trends in incidence increased faster than that of mortality since 1995. Thus, the better survival of PRL observed in our study since 2000 may reflect improved diagnostics in the modern era. Besides, the introduction of rituximab may also account for this improvement in prognosis, as the outcomes of patients with CD20-positive B lymphoma were significantly improved by the targeted therapy of rituximab^9,26–29^.

PRL is usually unilateral, whereas it can also be bilateral^2^. Here, we identified 7.9% of the cases with bilateral involvement with a slight predominance of left involvement. Previous studies suggested that PRL generally appeared to be bilateral in patients who were younger; these studies also showed a shorter survival time with bilateral PRL than with unilateral PRL^2^. In our study, patients younger than 18 also had a higher percentage of bilateral involvement. However, we did not observe a survival difference between unilateral and bilateral PRL.

Our data suggested that NHL constituted the majority of PRL, and the most prevalent subtype was DLBCL. HL accounted for only 0.7% of all lymphomas in our study. As an indolent type, MZL was associated with a good prognosis with a 1-year RS rate of 94% and a 5-year RS rate of 87%, while T-cell or NK-cell lymphoma revealed particularly poor outcomes, with a 1-year RS of 51%. We did not find a significant difference in survival in patients with renal DLBCL compared with nodal DLBCL.

The role of surgery in PRL is controversial, and evidence is based only on case series or literature reviews^9,30,31^. Belbaraka R *et al*. recommended chemotherapy regimens of R-CHOP to manage PRL rather than surgery^30^. However, Chen X. *et al*. reported a better outcome for patients who received surgical treatment^2^. In the current study with a large population, we observed that surgical treatment was associated with a significant improvement in survival. The use of radiation in PRL is also poorly studied. Here, we did not find a survival advantage for patients who received radiotherapy.

Our study has several limitations. First, the diagnosis of PRL has been controversial^2^. To reduce the risk of including secondary renal involvement of terminal systemic or adjacent lymphoma, our study focused on a diagnosis of lymphoma and a primary location in the kidney with no history of prior cancer diagnosis, as defined by the ICD-O-3. Second, there is a lack of treatment information on chemotherapy from the SEER database. It is not possible to know the actual proportions of patients who received chemotherapy, and such data are important because chemotherapy plays an important role in the management of lymphoma. Third, the SEER database does not provide other objective prognostic factors (such as the IPI, serum lactate dehydrogenase level) or other laboratory parameters in order to perform further analysis.

Nevertheless, based on a large population, the SEER database remains a valuable source for studying such rare kinds of cancers despite these limitations. This population-based approach can normalize the outcome analysis from potential selection biases, standardize both category and outcome definitions, and provide reasonable statistical utility to identify prognostic factors.

## Conclusion

In this study, we adopted a population-based dataset to examine the incidence, clinical characteristics and factors associated with survival of an extremely rare disease. The incidence rate of PRL is increasing over time. Older age, male gender, diagnosis before 2000, advanced stage, not receiving treatment with surgery, and DLBCL type or T/NK cell lymphoma type were associated with worse survival of patients with PRL in the multivariate analyses.

## Supplementary information

Supplementary materials

## Data Availability

The data is available on the Surveillance, Epidemiology, and End Results (SEER, http://seer.cancer.gov) database.
