# Supplementary material for: Primary renal lymphoma: a population-based study in the United States, 1980–2013
Source: Sci Rep. 2019 Oct 22;9:15125. doi: 10.1038/s41598-019-51635-6 (PMC6805900; doi:10.1038/s41598-019-51635-6)
Supplement: Supplementary file 1 — Supplementary materials [file 41598_2019_51635_MOESM1_ESM.pdf]

Primary renal lymphoma: a population-based study in the United States, 1980-2013

Jiayuan Chen, Jiangtong Peng， Yongqiang Zheng, Sen Li, Pengcheng Yang, Xiangyi Wu, He Tian, Hui Liu, Shengli Yang, Wenjing Wang, Bian Wu

Supplementary materials

Supplementary materials legends

Supplementary Figure 1. Kaplan–Meier estimate of relative survival (RS) and disease-specific survival (DSS) of patients with primary renal lymphoma (PRL) by different clinical factors.

Supplementary Figure 2. Kaplan–Meier estimate of relative survival (RS) and disease-specific survival (DSS) of patients with primary renal diffuse large B-cell lymphoma (DLBCL) vs. primary nodal DLBCL.

Supplementary Table 1. Vital status and Causes of death in patients with primary renal lymphoma (PRL)

Supplementary Table 2. Laterality of primary renal lymphoma (PRL) in patients younger than 18 vs. patients aging at 18 or older

Supplementary Table 3. Demographic characteristics and incidence rates of patients with primary renal lymphoma (PRL) by different sex in SEER-9 database

Supplementary Table 4. Annual incidence rate of primary renal lymphoma (PRL) in SEER-9 database

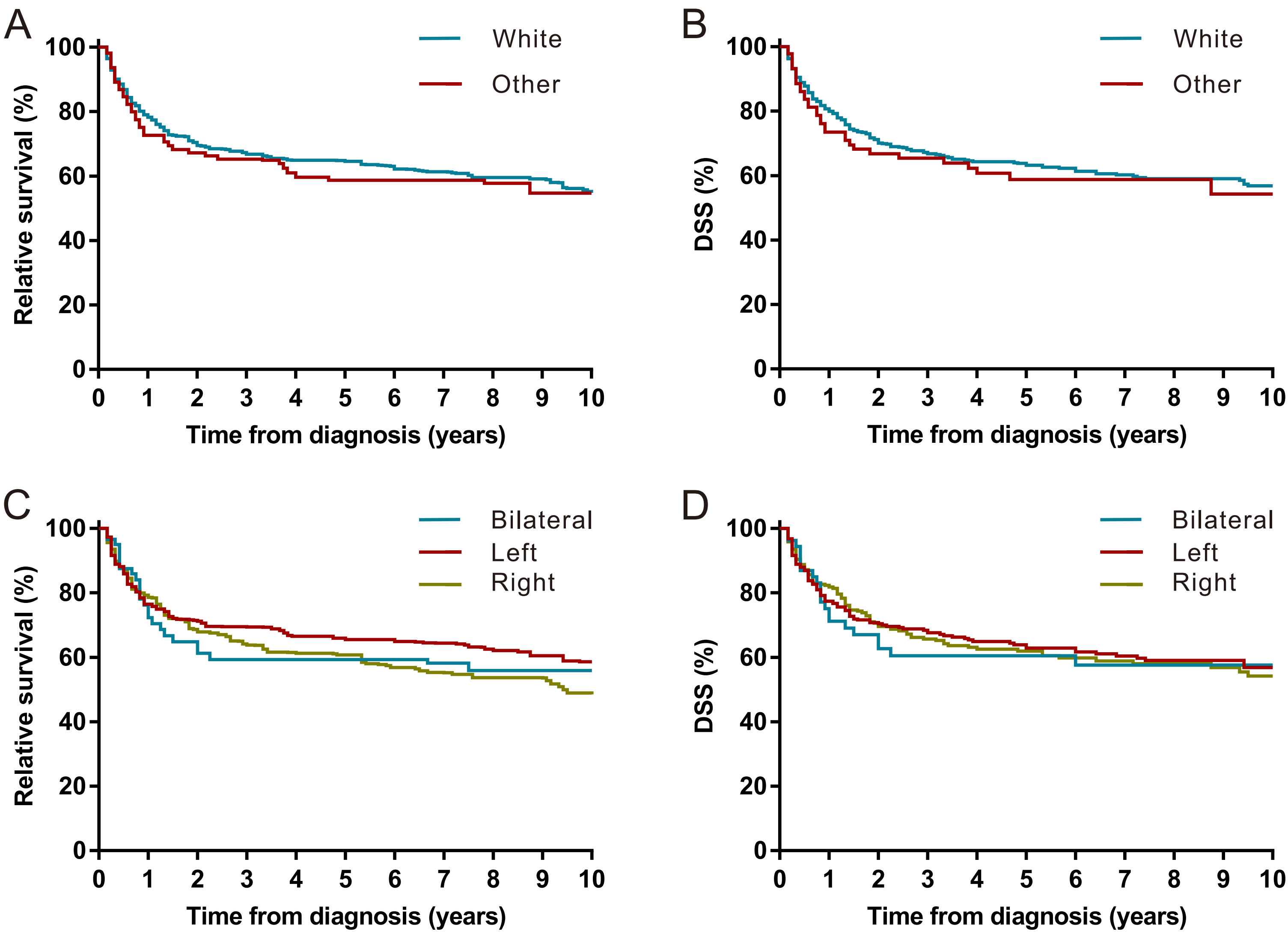

Supplementary Figure 1. Kaplan–Meier estimate of relative survival (RS) and disease-specific survival (DSS) of patients with PRL by different clinical factors. (A) RS by race; (B) DSS by race; (C) RS by laterality; (D) DSS by laterality.

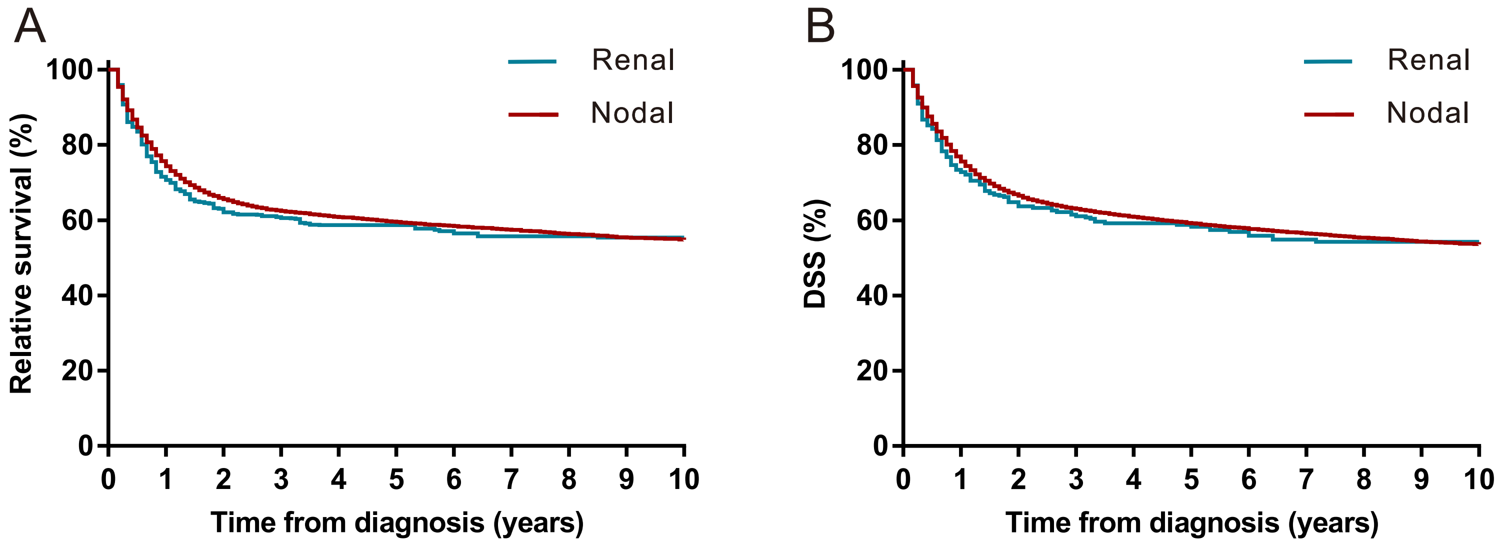

Supplementary Figure 2. Kaplan–Meier estimate of relative survival (RS) and disease-specific survival (DSS) of patients with primary renal diffuse large B-cell lymphoma (DLBCL) vs. primary nodal DLBCL. (A) RS; (B)DSS.

Supplementary Table 1. Vital status and Causes of death in patients with PRL.

Cause of death	No. (%)
All death	423 (100%)
PRL	253 (59.8%)
Cardiovascular diseases	46 (10.9%)
Other malignant cancers	45 (10.6%)
Infectious diseases	32 (7.6%)
Renal diseases	7 (1.7%)
Alzheimer’s disease (ICD-9 and 10 only)	4 (0.9%)
Diabetes mellitus	4 (0.9%)
Accidents and adverse effects	4 (0.9%)
Chronic obstructive pulmonary disease	3 (0.7%)
Chronic liver disease and cirrhosis	1 (0.2%)
Symptoms, signs and ill-defined conditions	2 (0.5%)
Other cause of death	19 (4.5%)
Unknown	3 (0.7%)

Supplementary Table 2. Laterality of PRL in patients younger than 18 vs. patients aging at 18 or older

Characteristics	No. of patients		p^a
	<18	≥18	%
Laterality			< 0.001
Bilateral	9 (39.1%)	48 (6.9%)	
Single	12 (52.2%)	620 (88.6%)	
Unknown or N/A	2 (8.7%)	32 (4.6%)	

N/A, not applicable.

[a]The chi-square test was used.

Supplementary Table 3. Demographic characteristics and incidence rates of patients with PRL by different sex in SEER-9 database

Characteristics	Demographic characteristics			Incidence rates		
	No. of patients	No. of Population	p^a	Age-Adjusted Rate	Standard Error	95% CI
All patients	436 (100%)	8.70E+08 (100%)		0.0528	0.0025	0.048-0.058
Sex			< 0.001			
Male	273 (62.6%)	4.27E+08 (49.1%)		0.0776	0.0048	0.069-0.088
Female	163 (37.4%)	4.43E+08 (50.9%)		0.0352	0.0028	0.030-0.041

[a]The chi-square test was used.

Supplementary Table 4. Annual incidence rate of PRL in SEER-9 database

Year	Overall incidence			Male incidence			Female incidence		
	Incidence rate[a]	SE	95% CI	Incidence rate[a]	SE	95% CI	Incidence rate[a]	SE	95% CI
**1980**	0.0201	0.0101	0.0054-0.052	0.0316	0.0183	0.0065-0.0958	0.0096	0.0096	0.0002-0.0535
**1981**	0.0127	0.0073	0.0026-0.0391	0.0267	0.0154	0.0055-0.0851	0.0001[b]	0.0001	0-0.0353
**1982**	0.0148	0.0086	0.003-0.0435	0.0219	0.0156	0.0026-0.0812	0.0091	0.0091	0.0002-0.0516
**1983**	0.0152	0.0088	0.0031-0.0444	0.0191	0.0135	0.0023-0.074	0.0099	0.0099	0.0002-0.0534
**1984**	0.0275	0.0113	0.01-0.0607	0.0181	0.0128	0.0022-0.0711	0.0337	0.0169	0.0091-0.088
**1985**	0.0295	0.0123	0.0105-0.0646	0.0228	0.0161	0.0028-0.0818	0.0368	0.019	0.0095-0.0953
**1986**	0.0304	0.0117	0.0119-0.0639	0.0533	0.0271	0.0141-0.1328	0.0158	0.0112	0.0019-0.0596
**1987**	0.0366	0.0131	0.0156-0.0728	0.0382	0.0194	0.0102-0.101	0.0331	0.0166	0.009-0.0859
**1988**	0.0268	0.012	0.0087-0.0613	0.0328	0.0241	0.0035-0.1081	0.0255	0.0148	0.0053-0.075
**1989**	0.0398	0.0135	0.0179-0.0763	0.0323	0.0165	0.0085-0.0884	0.0400	0.018	0.0128-0.0946
**1990**	0.0433	0.0146	0.0195-0.0819	0.0964	0.0327	0.0434-0.1833	0.0001[b]	0.0001	0-0.031
**1991**	0.0501	0.0152	0.0248-0.0896	0.0850	0.0335	0.0325-0.1746	0.0304	0.0152	0.0083-0.0793
**1992**	0.0351	0.0125	0.015-0.0692	0.037	0.0188	0.0098-0.0963	0.0338	0.017	0.0091-0.0861
**1993**	0.0431	0.0137	0.0206-0.0792	0.0764	0.0273	0.0326-0.151	0.0153	0.0108	0.0019-0.0561
**1994**	0.0593	0.016	0.0323-0.0996	0.0938	0.0302	0.0443-0.1734	0.0285	0.0143	0.0078-0.0745
**1995**	0.0505	0.0146	0.026-0.088	0.0537	0.022	0.0197-0.1171	0.0495	0.0203	0.0181-0.107
**1996**	0.0164	0.0082	0.0045-0.0419	0.0242	0.0178	0.0026-0.082	0.0145	0.0102	0.0018-0.0533
**1997**	0.0499	0.0144	0.0257-0.0868	0.0821	0.0276	0.0373-0.1556	0.0215	0.0125	0.0044-0.0638
**1998**	0.0688	0.0167	0.0401-0.1099	0.1120	0.0329	0.0572-0.1956	0.0353	0.0158	0.0114-0.0831
**1999**	0.0795	0.0178	0.0485-0.1227	0.1011	0.0294	0.0519-0.177	0.0561	0.0200	0.024-0.1115
**2000**	0.0500	0.0139	0.0266-0.0854	0.0745	0.0251	0.0337-0.142	0.0305	0.0152	0.0083-0.0774
**2001**	0.0699	0.0165	0.0414-0.1104	0.0974	0.0299	0.0479-0.1743	0.0513	0.0194	0.0206-0.1055
**2002**	0.0616	0.0155	0.0351-0.1	0.0741	0.0249	0.0336-0.1409	0.0507	0.0192	0.0203-0.1044
**2003**	0.0766	0.0172	0.0467-0.1184	0.1101	0.0319	0.0567-0.1908	0.0547	0.0194	0.0236-0.1084
**2004**	0.0558	0.0145	0.0311-0.0921	0.0800	0.0271	0.0361-0.1512	0.041	0.0167	0.015-0.0897
**2005**	0.0605	0.0152	0.0344-0.0982	0.0971	0.0295	0.0482-0.1725	0.0329	0.0148	0.0106-0.078
**2006**	0.0772	0.0166	0.0481-0.1173	0.1118	0.0316	0.0587-0.1911	0.0571	0.0191	0.026-0.1097
**Continued**									

Year	Overall incidence			Male incidence			Female incidence		
	Incidence rate[a]	SE	95% CI	Incidence rate[a]	SE	95% CI	Incidence rate[a]	SE	95% CI
**2007**	0.0631	0.0151	0.0371-0.1004	0.0926	0.0274	0.047-0.163	0.0379	0.0158	0.0135-0.0845
**2008**	0.0802	0.0173	0.0499-0.1218	0.0730	0.0249	0.0327-0.139	0.0876	0.0245	0.0464-0.1504
**2009**	0.0803	0.0166	0.0511-0.1202	0.1489	0.0349	0.0886-0.2335	0.0253	0.0114	0.0081-0.0629
**2010**	0.0896	0.0178	0.0583-0.1318	0.1598	0.0378	0.0945-0.2508	0.0447	0.0159	0.0192-0.0905
**2011**	0.0586	0.0141	0.0344-0.0935	0.0865	0.0265	0.0426-0.1547	0.0399	0.0153	0.0157-0.0846
**2012**	0.0473	0.0124	0.0262-0.0788	0.0572	0.0204	0.0244-0.1132	0.0438	0.0169	0.0172-0.0918
**2013**	0.0656	0.0142	0.0408-0.1003	0.1008	0.0275	0.0543-0.1702	0.0398	0.0142	0.0170-0.0818

SE, standard error; NR, not reached.

[a]Incidence rates were age-adjusted to the 2000 US standard population and expressed per 100,000 person-years.

[b]The values equal to zero when there were no cases reported were modified to a small positive constant (e. g. 0.0001), according to the statistical transformation.

Supplementary Table 5. Annual mortality rate of PRL in SEER-9 database

Year	Overall mortality rate			Male mortality rate			Female mortality rate		
	Incidence rate[a]	SE	95% CI	Incidence rate[a]	SE	95% CI	Incidence rate[a]	SE	95% CI
**1980**	0.0257	0.0115	0.0083-0.0601	0.0216	0.0153	0.0026-0.0813	0.0273	0.0158	0.0056-0.081
**1981**	0.0085	0.006	0.001-0.033	0.0185	0.0131	0.0022-0.0736	0.0001 [b]	0.0001	0-0.0353
**1982**	0.0001[b]	0.0001	0-0.0188	0.0001[b]	0.0001	0-0.0438	0.0001 [b]	0.0001	0-0.0348
**1983**	0.0157	0.0091	0.0032-0.0454	0.0215	0.0153	0.0026-0.0798	0.0099	0.0099	0.0002-0.0534
**1984**	0.0147	0.0086	0.0029-0.0433	0.0353	0.0218	0.0062-0.1055	0.0001 [b]	0.0001	0-0.0339
**1985**	0.0104	0.0074	0.0012-0.0368	0.0001 [b]	0.0001	0-0.0416	0.0184	0.013	0.0022-0.0661
**1986**	0.0148	0.0088	0.0028-0.0436	0.0280	0.0228	0.002-0.102	0.0092	0.0092	0.0002-0.0504
**1987**	0.0183	0.0094	0.0048-0.0475	0.0203	0.0147	0.0023-0.0756	0.0150	0.0106	0.0018-0.0575
**1988**	0.0328	0.0125	0.013-0.0677	0.0706	0.0307	0.0239-0.1558	0.0087	0.0087	0.0002-0.0485
**1989**	0.0144	0.0084	0.0029-0.0417	0.0001 [b]	0.0001	0-0.0386	0.0238	0.0138	0.0049-0.0711
**1990**	0.0198	0.0089	0.0063-0.0474	0.0324	0.0163	0.0087-0.0876	0.0078	0.0078	0.0002-0.0453
**1991**	0.0332	0.0126	0.0132-0.0679	0.0647	0.0301	0.0198-0.1482	0.0160	0.0113	0.0019-0.0584
**1992**	0.0207	0.0094	0.0066-0.0488	0.0258	0.0152	0.0051-0.0785	0.0142	0.01	0.0017-0.0538
**1993**	0.0448	0.0142	0.0214-0.082	0.0752	0.0295	0.029-0.1552	0.0218	0.0126	0.0045-0.0653
**1994**	0.0221	0.0099	0.0072-0.0512	0.0334	0.0194	0.0068-0.0939	0.0140	0.0099	0.0017-0.0526
**1995**	0.0472	0.0143	0.0235-0.0843	0.0586	0.0244	0.021-0.1272	0.0376	0.017	0.0121-0.0886
**1996**	0.0330	0.0117	0.0142-0.0649	0.0527	0.0216	0.0193-0.115	0.0163	0.0116	0.0019-0.0579
**1997**	0.0460	0.0139	0.0229-0.0819	0.0798	0.0289	0.0337-0.1566	0.0206	0.0119	0.0042-0.0617
**1998**	0.0327	0.0116	0.0141-0.0641	0.0565	0.0232	0.0206-0.1216	0.0125	0.0088	0.0015-0.0481
**1999**	0.0520	0.0144	0.0277-0.0888	0.0782	0.0262	0.0355-0.1482	0.0274	0.0138	0.0074-0.0715
**2000**	0.0273	0.0103	0.011-0.0561	0.0513	0.0211	0.0186-0.1116	0.0063	0.0063	0.0002-0.038
**2001**	0.0594	0.0154	0.0332-0.0978	0.0967	0.0294	0.048-0.1724	0.0273	0.0137	0.0074-0.071
**2002**	0.0506	0.014	0.0269-0.0863	0.0456	0.0209	0.0143-0.1056	0.0537	0.0191	0.023-0.1069
**2003**	0.0260	0.0099	0.0104-0.0536	0.0314	0.0182	0.0064-0.0868	0.0256	0.0128	0.0069-0.0672
**2004**	0.0303	0.0108	0.013-0.0597	0.0663	0.0254	0.0263-0.1346	0.0053	0.0053	0.0001-0.0347
**2005**	0.0609	0.0153	0.0347-0.0989	0.1108	0.0335	0.0552-0.195	0.0335	0.0152	0.0107-0.0793
**2006**	0.0540	0.0141	0.0301-0.0894	0.0648	0.0248	0.0257-0.1316	0.0522	0.0185	0.0225-0.1039
**Continued**									

Year	Overall mortality rate			Male mortality rate			Female mortality rate		
	Incidence rate[a]	SE	95% CI	Incidence rate[a]	SE	95% CI	Incidence rate[a]	SE	95% CI
**2007**	0.0401	0.0117	0.0205-0.0707	0.0613	0.0235	0.0243-0.1251	0.0265	0.0119	0.0085-0.0653
**2008**	0.0296	0.01	0.0134-0.0569	0.0289	0.0149	0.0075-0.0756	0.0298	0.0137	0.0093-0.0721
**2009**	0.0254	0.0091	0.0109-0.0508	0.0411	0.0186	0.0132-0.0948	0.0141	0.0081	0.0029-0.0462
**2010**	0.0641	0.0149	0.0383-0.1006	0.1350	0.0341	0.0766-0.2184	0.0150	0.0087	0.003-0.0482
**2011**	0.0537	0.0136	0.0304-0.0877	0.1149	0.0311	0.0622-0.1923	0.0101	0.0072	0.0012-0.041
**2012**	0.0468	0.0127	0.0253-0.079	0.0888	0.0271	0.0438-0.1582	0.0211	0.0122	0.0044-0.061
**2013**	0.0516	0.0127	0.0298-0.0835	0.0805	0.0247	0.0396-0.1447	0.0334	0.014	0.0119-0.0755

SE, standard error; NR, not reached.

[a]Mortality rates were age-adjusted to the 2000 US standard population and expressed per 100,000 person-years.

[b]The values equal to zero when there were no cases reported were modified to a small positive constant (e. g. 0.0001), according to the statistical transformation.
